# Improving the Practice of Delayed Cord Clamping at American University of Beirut Medical Center (AUBMC): A Quality Improvement Project

**DOI:** 10.3390/children13091250

**Published:** 2026-09-15

**Authors:** Mohamad Hammoud, Hussein Hussein, Safaa AlSarout

**Affiliations:** 1 Department of Pediatrics and Adolescent Medicine, Division of Neonatology, American University of Beirut Medical Center, Beirut P.O. Box 11-0236, Lebanon; 2 Department of Neonatology and Neonatal Medicine, Hôpital René-Dubos (NOVO Hospital), 95300 Pontoise, France; 3 Department of Neonatology and Neonatal Medicine, Centre Hospitalier Universitaire d’Orléans, 45067 Orléans, France; safaa.alsarout@chu-orleans.fr

**Keywords:** delayed cord clamping, quality improvement, PDSA, SQUIRE 2.0, documentation, neonatal care

## Abstract

**Highlights:**

**What are the main findings?**

- Documented DCC increased between the initial and highest two-month summaries; however, the 80% target was not reached.
- The highest monthly rates were observed in January and February 2025, reaching 67.4% and 66.7%, respectively.
- Available aggregate records did not establish adherence to a measured clamping interval of at least 60 s or demonstrate improved neonatal outcomes.

**What are the implications of the main findings?**

- Implementation requires reliable timing, clear responsibilities and consistent documentation alongside education.
- Future audits should distinguish clinical exceptions, missed opportunities and missing timing information.
- Sustainability and neonatal safety require longer follow-up with complete, standardized balancing measures.

**Abstract:**

**Background:** Delayed cord clamping (DCC) is recommended for infants who do not require immediate resuscitation, but reliable implementation requires coordination across delivery-room teams. This quality-improvement project aimed to increase DCC uptake at AUBMC to 80%, promote clamping at ≥60 s and improve staff knowledge. **Methods:** Monthly aggregate records from January 2024 to February 2025 were analyzed in a single-center, uncontrolled QI evaluation. A multidisciplinary intervention package, reported to have commenced in September 2024, combined education, timing support, protocol/checklist development and electronic documentation initiatives. The primary measure was the proportion of tabulated deliveries classified as DCC, rather than independently verified adherence to a measured clamping interval. Counts, Wilson 95% confidence intervals (CIs) and an exploratory run chart were used. **Results:** The monthly dataset contained 956 deliveries, including 417 classified as DCC (43.6%). The median monthly proportion increased from 42.24% in January–February 2024 to 67.03% in January–February 2025. The 80% target was not achieved. An exploratory above-median sequence began before the reported intervention start, limiting attribution. Duration data and adequately characterized knowledge and safety datasets were unavailable for confirmatory analysis. **Conclusions:** The project showed partial improvement in documented DCC, with the highest rates observed in January–February 2025. More reliable measurement, explicit delivery-room responsibilities and continued audit are needed before concluding that duration-based adherence, safety or sustainability improved.

## 1. Introduction

Delayed cord clamping supports placental transfusion and improves early hematologic outcomes and iron status in term infants [1]. In preterm infants, contemporary individual-participant-data meta-analysis provides high-certainty evidence of lower mortality before discharge with deferred rather than immediate clamping [2]. A companion network meta-analysis further examined the relationship between deferral duration and outcomes [3]. The 2025 AHA/AAP guidelines and ninth-edition Neonatal Resuscitation Program materials support deferral for at least 60 s when immediate resuscitation is not required [4,5]. These updated publications postdate the observation period and provide current context rather than a retrospective basis for the original intervention.

Despite the established recommendations, DCC depends on multiple coordinated actions at birth: agreeing on eligibility, assigning responsibility for timing, assessing the infant, maintaining warmth and recording the actual interval. Competing clinical tasks and differing thresholds for initiating resuscitation may lead to early clamping. During cesarean delivery, surgical workflow and access to the infant add coordination requirements. These are plausible implementation mechanisms; the present project did not quantify their individual effects.

AUBMC reported approximately 800 births annually. The initial January–February 2024 monthly DCC proportions were 44.2% and 40.3%, indicating a local gap between recommendations and recorded practice. The project team identified timing support, awareness, workflow and documentation as targets for improvement. The rationale was that education would address knowledge gaps, while explicit timing and documentation processes would help translate knowledge into routine care. Provider hesitancy was reported qualitatively, but the extent of resistance and its distribution across professional groups were not measured.

**Figure 1:**
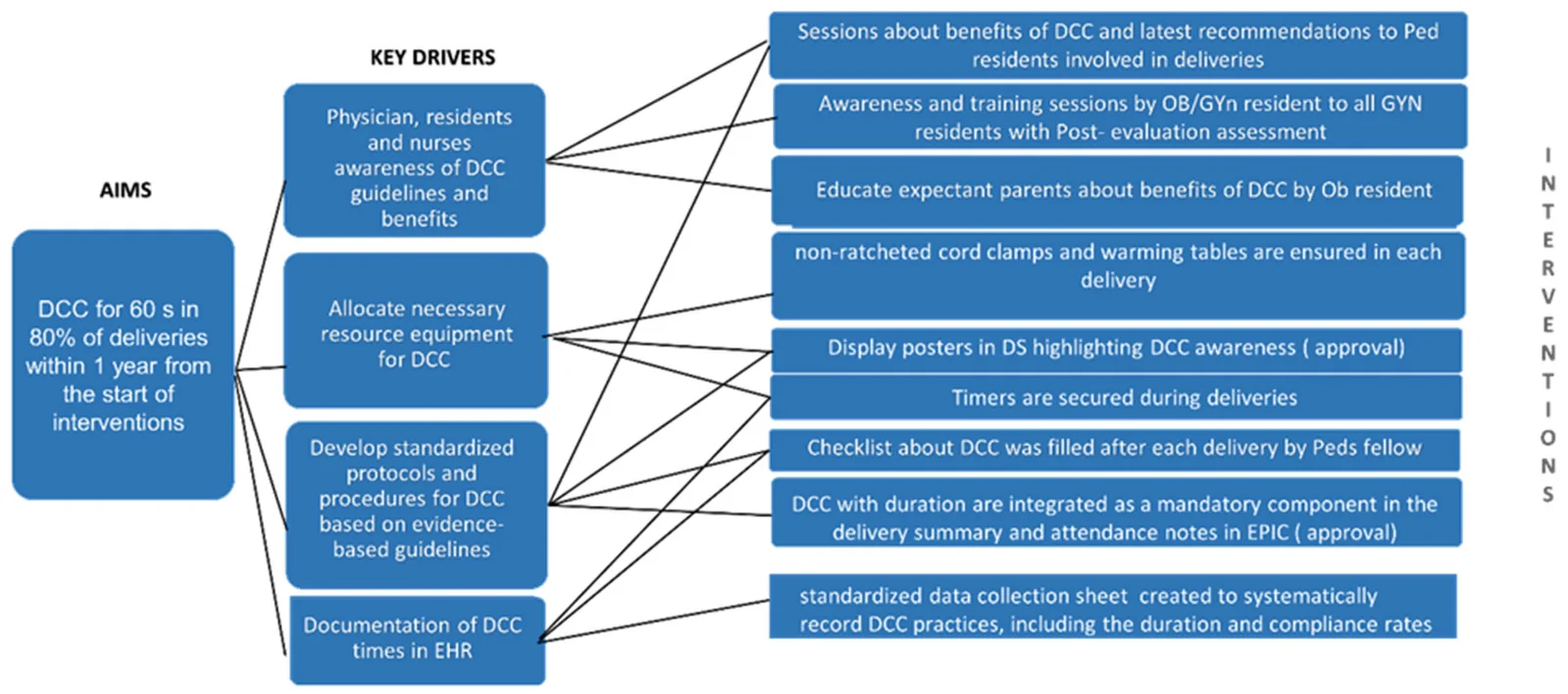
Key driver diagram guiding the delayed cord clamping quality-improvement workflow at AUBMC. The diagram summarizes the project aim and the principal drivers addressed through education, standardized workflow, timing support, documentation and feedback.

The project aimed to reach 80% DCC uptake, promote a clamping interval of at least 60 s and improve staff knowledge. This report evaluates documented uptake and implementation limitations in term and late preterm infants. Its contribution is to identify measurement and workflow gaps remaining after a relatively simple intervention package; it does not test the clinical efficacy of DCC or establish effectiveness in very preterm infants. The original key driver diagram summarizes the intended changes (Figure 1).

## 2. Materials and Methods

### 2.1. Design and Setting

This single-center quality-improvement (QI) project was conducted at the American University of Beirut Medical Center (AUBMC) and evaluated delayed cord clamping (DCC) practices from January 2024 to February 2025. January–February 2024 served as the initial reference period, and the multidisciplinary improvement workflow was introduced during the study period. The project was organized according to a Plan–Do–Study–Act (PDSA) approach and is reported in accordance with the SQUIRE 2.0 framework [6].

### 2.2. Population and Outcome Measures

The target population comprised term and late-preterm infants (≥34 weeks) delivered at AUBMC. Monthly delivery totals were used as the analytic denominator. The primary process measure was the proportion of deliveries classified as having received DCC. The project target was to increase DCC uptake to 80% and to promote a clamping interval of at least 60 s when clinically appropriate.

Documented DCC was calculated monthly as the number of deliveries classified as DCC divided by the corresponding monthly total. Additional measures included delivery characteristics, one-minute Apgar score, need for resuscitation, presence of timer personnel, thermal-care documentation, resident knowledge scores, NICU admission and phototherapy.

For the project protocol, clamping before 30 s was considered early clamping, 30–59 s was below the intended target, and an interval of at least 60 s represented target attainment. Monthly DCC classification was analyzed as the principal available measure of implementation.

### 2.3. Improvement Intervention and Delivery-Room Workflow

The intervention was multidisciplinary and involved neonatology, obstetric and nursing teams. The key driver diagram (Figure 1) guided a workflow focused on staff awareness, standardized delivery-room practice, reliable timing and documentation. Education was provided to pediatric and obstetric residents and addressed the rationale, benefits, possible complications and practical steps of DCC. Educational support included lectures and discussion, together with handouts, posters and a delivery-room checklist.

The delivery-room protocol emphasized starting the timing process at complete birth, maintaining neonatal thermal care, assessing the infant during the delay and documenting the clamping interval. Timers were introduced to support consistent measurement, and the protocol/checklist was used to standardize the sequence of care. Documentation of DCC in the electronic health record was incorporated into the improvement strategy, with the monthly review of DCC rates used to provide feedback and guide subsequent improvement actions.

### 2.4. PDSA Approach and Education Assessment

The PDSA cycle addressed four linked implementation domains: awareness and education, timing, standardized workflow and documentation. During the Do phase, the team implemented education, timing support and the DCC protocol/checklist. During the Study phase, monthly DCC proportions and resident knowledge scores were reviewed. The Act phase focused on reinforcing timing responsibility, documentation and continued audit in response to observed performance.

Resident knowledge was assessed before and after education using scores addressing DCC procedures, benefits and possible complications. Mean pre- and post-education scores were summarized by postgraduate year (PGY) and used as a descriptive educational measure.

### 2.5. Statistical Analysis

The analysis included 14 monthly observations. Monthly and pooled DCC proportions were calculated with Wilson 95% confidence intervals (CIs). Medians were calculated from unrounded monthly proportions, whereas pooled proportions were based on summed numerators and denominators. An exploratory run chart used the January–February 2024 median as the initial fixed reference. A shift was defined as at least six consecutive points on one side of the median and a trend as at least five consecutive increasing or decreasing points [7]. A sensitivity analysis used the January–August 2024 median before the reported intervention rollout. Delivery characteristics, Apgar scores, balancing measures and resident knowledge scores were analyzed descriptively; no adjusted causal model was fitted.

### 2.6. Ethical Considerations

The Quality Advisory Council at AUBMC reviewed and approved the project, titled “Increasing the Practice of Delayed Cord Clamping in Newborns,” as a quality improvement initiative and determined that institutional review board (IRB) approval was not required. The hospital ethics committee also agreed to the conduct of the project.

## 3. Results

The study included 956 deliveries, of which 417 were classified as DCC and 539 were not classified as DCC. Monthly denominators ranged from 46 to 85 deliveries. Overall documented DCC uptake was 43.6% (95% CI 40.5–46.8%). The lowest monthly rate was observed in May 2024 (22/81, 27.2%), while the highest rate occurred in January 2025 (31/46, 67.4%). February 2025 had the highest absolute number of DCC cases (42/63, 66.7%).

The median monthly DCC proportion increased from 42.24% in January–February 2024 to 67.03% in January–February 2025. The corresponding pooled proportions were 59/139 (42.4%; 95% CI 34.5–50.8%) and 73/109 (67.0%; 95% CI 57.7–75.1%). January–February 2025 represented the highest two-month performance during the observation period.

From January to August 2024, the documented DCC was 216/568 (38.0%; 95% CI 34.1–42.1%). From September 2024–February 2025, it was 201/388 (51.8%; 95% CI 46.8–56.7%), corresponding to a descriptive increase of 13.8 percentage points (Table 1).

Among DCC-classified records, 44.2% were vaginal births, 51.0% were elective cesarean deliveries and 4.8% were urgent cesarean deliveries. In the comparison group, the corresponding proportions were 24.7%, 64.2% and 11.1%, respectively. Gestational-age distributions and additional delivery characteristics are presented in Table 2.

Mean one-minute Apgar scores were 7.69 in the DCC-classified group and 7.14 in the comparison group. The reported need for resuscitation was 6.7% and 6.2%, respectively. Timer personnel were recorded in 15.4% of DCC-classified records and 7.4% of comparison records, while thermal-care documentation was recorded in 94.2% and 98.8%, respectively.

Values are percentages, except for the mean Apgar score. Percentages are presented as recorded in the study dataset.

**Figure 2:**
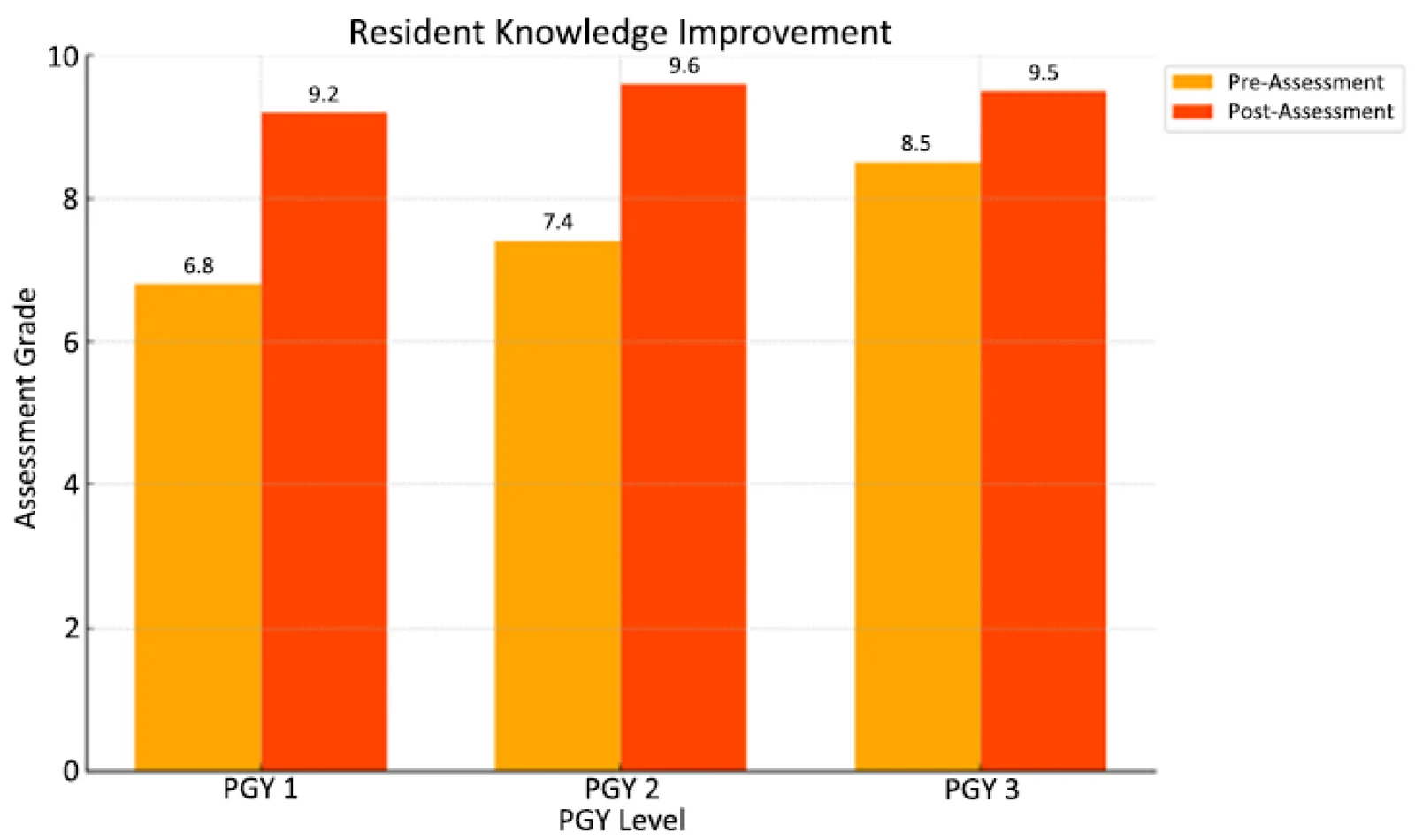
Resident knowledge scores before and after DCC education, by postgraduate year (PGY). Bars represent the recorded group mean scores.

Mean resident knowledge scores increased after education in all three postgraduate-year groups: from 6.8 to 9.2 in PGY-1, from 7.4 to 9.6 in PGY-2 and from 8.5 to 9.5 in PGY-3 residents (Figure 2).

**Figure 3:**
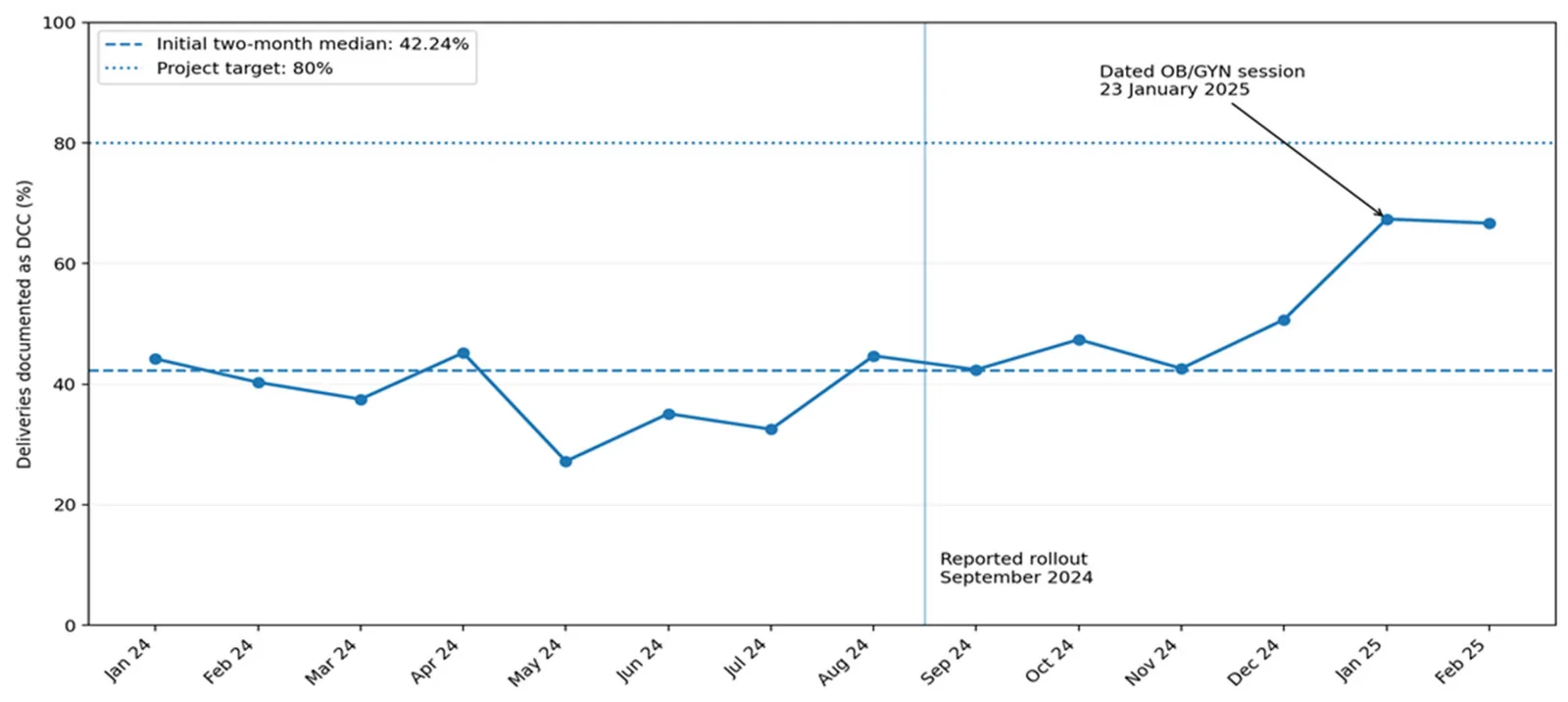
Monthly documented DCC, January 2024–February 2025, based on Table 1. The fixed reference line represents the January–February 2024 median (42.24%), and the project target is 80%.

The run chart showed seven consecutive monthly observations above the initial fixed median of 42.24% from August 2024 to February 2025, meeting the predefined exploratory shift rule. The same seven observations were above the alternative January–August median of 38.91%. No five-point monotonic trend was identified (Figure 3).

**Figure 4:**
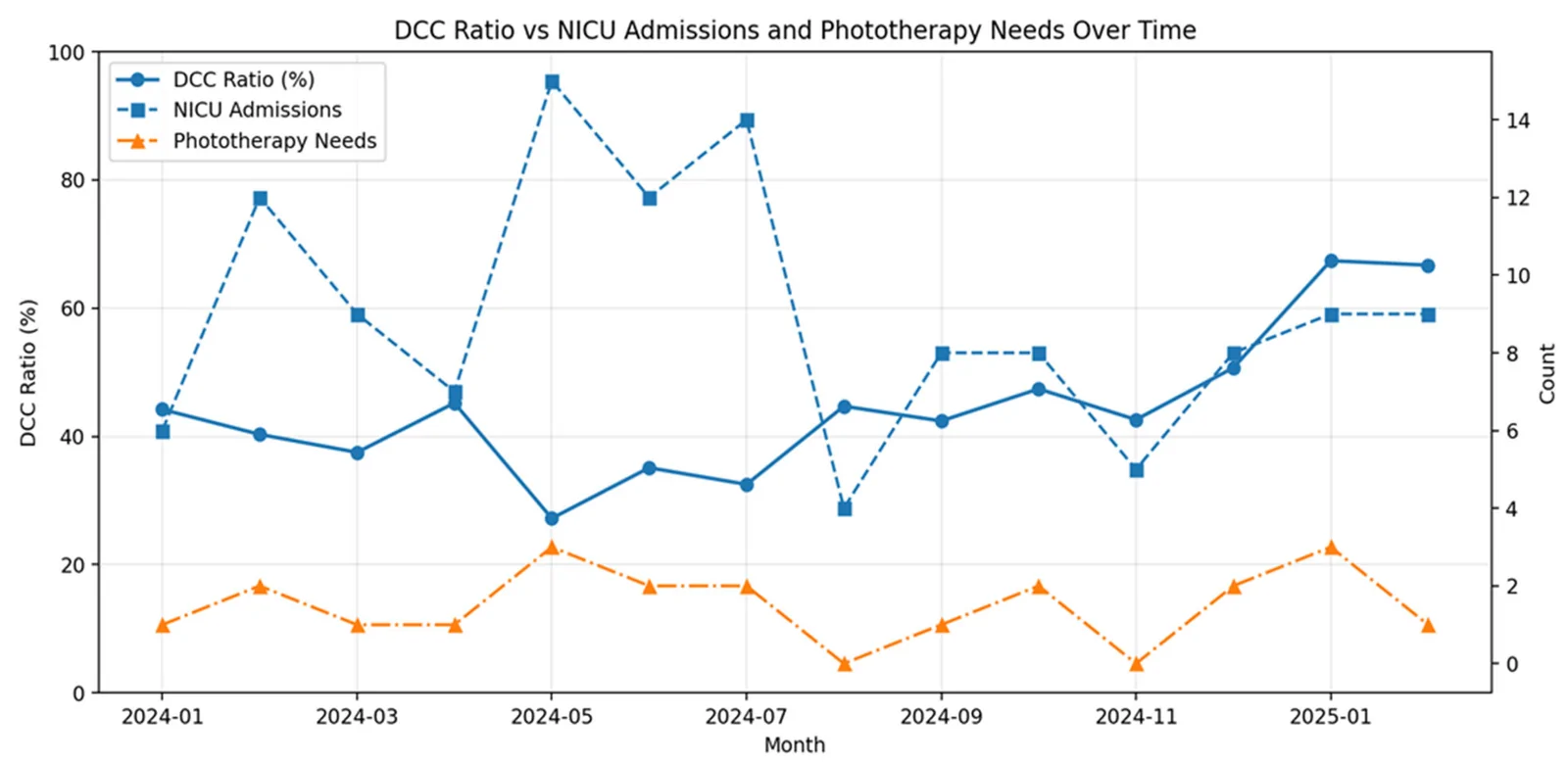
Monthly DCC percentages and reported balancing measures. DCC is displayed on the left-hand percentage axis; NICU admissions and phototherapy are displayed as counts on the right-hand axis.

Figure 4 displays the monthly DCC percentage together with the reported monthly counts of NICU admissions and phototherapy as balancing measures. DCC is shown on the left-hand percentage axis, while NICU admissions and phototherapy are shown on the right-hand count axis. Operational observations during implementation included inconsistent timer coverage, variable staff awareness, provider hesitancy and incomplete documentation. These observations informed continued reinforcement of timing responsibility, workflow standardization and documentation during the improvement process.

## 4. Discussion

This project showed a temporal increase in documented DCC but did not achieve the 80% target. The peak two-month median of 67.03% in January–February 2025 should be interpreted as partial implementation, particularly in a population described as term and late preterm. The contribution is an account of implementation and measurement gaps; the results do not demonstrate improved neonatal health or independently verified duration-target attainment.

Education formed one component of the broader intervention package and was accompanied by timing support, a standardized protocol/checklist, documentation initiatives and monthly feedback. The increase in resident knowledge scores is consistent with improved short-term knowledge after education, although the educational component cannot be separated from other contemporaneous changes in practice. Similarly, improvements in recorded DCC may reflect both changes in clinical behavior and improvements in documentation.

Cesarean delivery deserves focused improvement work. The reported group distributions suggest unequal representation of delivery modes, but they cannot be converted into mode-specific success rates without counts. Surgical priorities, infant access, sterile-field thermal care and rapid escalation during urgent delivery are plausible barriers requiring direct audit. Cesarean delivery by itself should not be equated with a contraindication. A recent cesarean-specific systematic review provides relevant evidence for discussing DCC in this setting [8]. An appropriate next step is separate reporting of vaginal, elective cesarean and emergency cesarean births, with documented clinical exceptions.

Continued multidisciplinary engagement is important for sustainability. Attending neonatologists, obstetricians, residents and nurses should participate directly in planning, training and review because senior clinical judgment and team coordination influence decisions about neonatal stabilization and the feasibility of DCC. Parent education may also support shared understanding of the delivery-room plan when clinically appropriate.

The project protocol provided a structured framework for DCC implementation, while subsequent evidence supports refinement of several practical elements. In vigorous term vaginal births, maternal abdominal/chest positioning can be used without requiring placental-level positioning [9], and palpable cord pulsations do not reliably indicate cessation of blood flow [10]. Current guidance also supports defined thermal targets and timely neonatal support [4,11]. Appendix A and Appendix B present an updated workflow and checklist for future improvement cycles.

The improvement observed during the later months of the study supports continued monthly audits, reinforcement of assigned timing responsibilities and review of clinical exceptions or missed opportunities. The exploratory run-chart shift beginning before the reported intervention rollout also indicates that temporal improvement should not be attributed to the QI package alone.

NICU admission and phototherapy were monitored as balancing measures and are presented descriptively in Figure 4. Interpretation of safety outcomes should remain cautious because the available aggregate data do not support adjusted comparisons between DCC and non-DCC groups. The difference in mean one-minute Apgar scores should likewise be interpreted descriptively, as the clinical condition can influence the decision to clamp the cord earlier.

Limitations include the single-center uncontrolled design, the relatively short initial reference period and the use of aggregate monthly data. The dataset did not allow independent verification of all eligibility and exclusion criteria, handling of multiple births, or delivery-level linkage of DCC classification with clamping duration. Exact elapsed clamping times were not available for analysis, so documented DCC could not be equated with verified attainment of the ≥60 s target. Detailed training attendance, the full resident knowledge questionnaire, score dispersion and paired individual results were unavailable, limiting formal analysis of the educational outcome. Delivery-characteristic percentages lacked complete n/N and missing-data information, and mode-specific DCC success rates could not be calculated. The balancing measures were available as reported monthly counts without complete event definitions, ascertainment windows or linked eligible denominators, and neonatal temperature data were unavailable; therefore, safety comparisons should remain descriptive. The intervention chronology and implementation intensity of individual components, including electronic documentation, were not available at a level that permitted attribution of effect to a specific component. In addition, the exploratory run-chart signal began before the reported September rollout, limiting causal interpretation. Costs and staff time were not measured, and longer-term sustainability beyond February 2025 was not assessed.

The next improvement cycle should test explicit allocation of a timer and neonatal assessment lead, supported by a brief pre-delivery discussion and a consistent recording field for elapsed seconds. A sample of records should be checked against direct observation. Monthly feedback should distinguish verified ≥60 s clamping, clinically justified early clamping, unexplained short intervals and unknown timing. Training should include attending obstetricians and neonatologists, residents and nurses, with simulation considered for cesarean and urgent scenarios. These are proposed actions, not completed interventions.

A subsequent cycle should prospectively collect temperature, resuscitation timing, NICU admission and phototherapy with defined denominators and review whether ≥80% eligible-birth uptake can be maintained over consecutive months. Very preterm infants require a separately designed pathway with appropriate thermal and respiratory support. Their inclusion is important in future work because mortality benefits are supported by strong trial synthesis [2,3], but the present study cannot establish transferability to that higher-risk population.

## 5. Conclusions

This single-center QI project showed partial improvement in documented DCC among term and late-preterm infants, although the 80% target was not achieved. Reliable timing, clearer delivery-room responsibilities and complete outcome measurement are needed to assess sustained duration-based adherence and safety.

## Figures and Tables

**Table 1:** Monthly documented DCC, January 2024–February 2025.

Month	DCC, n	Not Classified as DCC, n	Total, n	DCC % (95% CI)
January 2024	34	43	77	44.2 (33.6–55.3)
February 2024	25	37	62	40.3 (29.0–52.7)
March 2024	21	35	56	37.5 (26.0–50.6)
April 2024	28	34	62	45.2 (33.4–57.5)
May 2024	22	59	81	27.2 (18.7–37.7)
June 2024	26	48	74	35.1 (25.2–46.5)
July 2024	26	54	80	32.5 (23.2–43.4)
August 2024	34	42	76	44.7 (34.1–55.9)
September 2024	36	49	85	42.4 (32.4–53.0)
October 2024	37	41	78	47.4 (36.7–58.4)
November 2024	20	27	47	42.6 (29.5–56.7)
December 2024	35	34	69	50.7 (39.2–62.2)
January 2025	31	15	46	67.4 (53.0–79.1)
February 2025	42	21	63	66.7 (54.4–77.1)
Total	417	539	956	43.6 (40.5–46.8)

CI, confidence interval. Wilson 95% confidence intervals are shown for monthly DCC proportions.

**Table 2:** Reported delivery characteristics by documented DCC status.

Characteristic	DCC Classified	Comparison Group
Gestational age		
Late preterm (34–<37 weeks)	6.7	14.8
Early term (37–<39 weeks)	18.3	16.0
Term (≥39 weeks)	75.0	67.9
Type of delivery		
Normal vaginal delivery	44.2	24.7
Elective cesarean section	51.0	64.2
Urgent cesarean section	4.8	11.1
Mean Apgar score at 1 min	7.69	7.14
Need for resuscitation	6.7	6.2
Presence of timer personnel	15.4	7.4
Presence of thermal wrap	94.2	98.8

## Data Availability

Monthly aggregate DCC counts are reported in Table 1. The availability of additional de-identified data is subject to institutional requirements and confirmation by the corresponding author.

## Appendix A. Proposed Updated DCC Workflow

This proposed workflow reflects current guidance and is intended for a future locally approved improvement cycle. It is not a retrospective description of care delivered during January 2024–February 2025.

***Preparation and responsibility***

Before birth, identify the neonatal assessment lead, obstetric lead, the person timing the interval, and the person recording it. The assessment lead determines neonatal escalation needs; the obstetric lead identifies maternal indications for intervention. The team should communicate any need to interrupt DCC immediately.

***Timing and initial care***

Start the timer at complete birth and aim for ≥60 s when immediate resuscitation is unnecessary. The neonatal clinician assesses breathing, tone and heart rate as indicated while providing drying and gentle stimulation. Do not routinely use suction; reserve suction for suspected airway obstruction [4].

***Position and warmth***

For vigorous term vaginal births, maternal abdominal/chest skin-to-skin positioning is an option while avoiding cord traction [9]. Maintain the delivery area at 23–25 °C and the infant at 36.5–37.5 °C [11]. For cesarean birth, agree on a secure position within the sterile field; use locally approved warming methods that preserve sterility. Record whether a warmer is used during intact-cord care or only after clamping.

***Interruption and escalation***

Persistent apnea/gasping or heart rate < 100/min after initial steps requires timely ventilation. If appropriate support cannot be provided with the cord intact, clamp and transfer without waiting for the target interval [4]. Major maternal instability/hemorrhage or disrupted placental circulation require urgent joint reassessment. Record the specific indication rather than “distress” alone.

***Completion***

Use measured elapsed time, not the disappearance of palpable cord pulsations, to document the interval [10]. Record actual seconds, the clinical reason for a shorter interval, whether timing is missing, and any support provided before and after clamping. Do not imply that a particular clamp design improves transfusion.

***Measurement for the next cycle***

Record delivery mode and urgency, clinical eligibility, timer coverage, clamping seconds, neonatal temperature/time/site, resuscitation start time, NICU admission and phototherapy within prespecified periods. Review clinical exceptions separately from missed opportunities; maintain an explicit missing-data category.

## Appendix B. Proposed Revised DCC Checklist

**Field**	**Record/Response**
Birth details	Local record identifier: ______ Date/time: ______ Gestational age: ______ Infant sex: ______
Delivery mode and urgency	Vaginal/elective cesarean/emergency cesarean
Pre-delivery briefing	DCC discussed: Yes/No Eligibility and any anticipated exception: ______
Assigned team	Neonatal assessment lead: ______ Obstetric lead: ______ Timer: ______ Recorder: ______
Timing readiness	Timer available and person assigned: Yes/No Timing started at complete birth: Yes/No/Unknown
Thermal preparation	Room 23–25 °C: Yes/No/Not measured Measured room temperature: ______ Dry covering/hat and approved warming method: ______
Position and cesarean sterility	Infant position without cord traction: ______ For cesarean birth, sterile warming method: ______ Warmer used before/after clamping/not used
Actual clamping interval	Elapsed seconds: ______/Unknown <30 s/30–59 s/≥60 s/Unknown
Reason for interval < 60 s	Specific neonatal or maternal indication: ______ Operational reason/unexplained omission/missing timing: ______
Assessment and support	Breathing/tone/heart rate assessment as indicated: ______ Support with cord intact and after clamping: ______ Ventilation start time, if needed: ______
Temperature and balancing measures	Infant temperature: ______°C Time/site: ______ NICU admission and indication: ______ Phototherapy: ______ Use prospectively defined ascertainment windows.
Documentation	Actual seconds, exceptions and unknown values entered in EHR: Yes/No Recorder and completion time: ______
